# The Advanced Reasoning Capabilities of Large Language Models for Detecting Contraindicated Options in Medical Exams

**DOI:** 10.2196/68527

**Published:** 2025-05-12

**Authors:** Yuichiro Yano, Mizuki Ohashi, Taiju Miyagami, Hirotake Mori, Yuji Nishizaki, Hiroyuki Daida, Toshio Naito

**Affiliations:** 1 Department of General Medicine, Juntendo University Faculty of Medicine, 2-1-1, Hongo, Bunkyo-Ku, Tokyo, 113-8421, Japan, 81 3-3813-3111; 2 AI Incubation Farm, Juntendo University Faculty of Medicine, Tokyo, Japan; 3 Division of Medical Education, Juntendo University School of Medicine, Tokyo, Japan; 4 Department of Cardiovascular Biology and Medicine, Juntendo University Graduate School of Medicine, Tokyo, Japan

**Keywords:** natural language processing, artificial intelligence, clinical reasoning, medical errors, large language model

## Abstract

Enhancing clinical reasoning and reducing diagnostic errors are essential in medical practice; OpenAI-o1, with advanced reasoning capabilities, performed better than GPT-4 on 15 Japanese National Medical Licensing Examination questions (accuracy: 100% vs 80%; contraindicated option detection: 87% vs 73%), though findings are preliminary due to the small sample size.

## Introduction

Diagnostic errors account for more than 8% of adverse medical events and up to 30% of malpractice claims [1]. Enhancing clinical reasoning could mitigate this [2], improving patient outcomes and potentially lowering legal liabilities. In September 2024, OpenAI introduced OpenAI-o1, a large language model (LLM) trained with reinforcement learning to enhance its complex “reasoning” [3,4]. Key enhancements include advanced attention mechanisms, refined training data and curation, and enhanced fine-tuning protocols [3,5]. However, it remains uncertain whether OpenAI-o1 can improve clinical reasoning and reduce diagnostic errors.

In the Japanese National Medical Licensing Examination (JNMLE), candidates must not only achieve high overall accuracy but also avoid selecting contraindicated options—errors that can lead to failure even if most answers are correct. Although prior studies indicate that ChatGPT-4 performs well on the JNMLE, it sometimes chooses contraindicated options [6]. We posited that OpenAI-o1 would exhibit superior reasoning compared to GPT-4 and hypothesized that it would more proficiently avoid contraindicated options.

## Methods

On October 10, 2024, we used 15 text-based JNMLE questions (from 2019 to 2024) that included contraindicated options (Multimedia Appendix 1). Questions with images were excluded due to OpenAI-o1’s inability to process visual data. We administered the questions to both GPT-4 and OpenAI-o1, with each model evaluated under the supervision of designated examiners (MO and TM).

The examination comprised 3 steps: (1) Japanese examination—select correct answers, (2) Japanese examination—identify contraindicated options, and (3) English examination—repeat steps 1 and 2 with translated questions. Translation used an automated system and was reviewed by bilingual clinical expert YY.

The responses from both models were recorded, and the results were evaluated based on the numbers of correct answers and correctly identified contraindicated options in both languages.

## Results

As shown in Multimedia Appendix 1, among the 15 questions, GPT-4 correctly answered 12 (80%) and identified 11 contraindicated options (73%) in Japanese. In English, GPT-4 correctly answered 13 questions (87%) and identified 11 contraindicated options (73%). OpenAI-o1 correctly answered 15 questions (100%) and identified 13 contraindicated options in Japanese (87%). Both GPT-4 and OpenAI-o1 had consistently equal or better performance in English than Japanese, especially for contraindicated options.

## Discussion

OpenAI-o1 had higher accuracy than GPT-4 and was better able to select contraindicated options on the JNMLE, particularly in English. However, this difference was minimal—only 1 of 15 questions showed improvement in English—indicating that language had little overall impact.

In medicine, avoiding contraindicated actions is crucial. While correct answers reflect basic medical knowledge, recognizing what should not be done requires advanced critical thinking and reasoning. Errors can lead to patient harm, lawsuits, or even license revocation. Here, OpenAI-o1 outperformed GPT-4 in identifying contraindicated actions. OpenAI-o1’s enhancements [3,5] and our finding of its superior reasoning ability suggest the importance of using LLMs with robust reasoning capabilities for medical licensing examinations and, by extension, in clinical practice, to safeguard patient safety and uphold high standards of care.

Our study is limited, first, by using only 15 questions, so these findings should be interpreted as preliminary and hypothesis-generating. Second, we used the models’ default settings without fine-tuning, prompt engineering, or chain-of-thought modifications, capturing their performance at only a specific time point. Third, we obtained a single response per query, which may not reflect the full variability of LLM outputs. Fourth, continuous model updates limit exact reproducibility. Fifth, only 2 of 15 questions showed discrepancies, limiting our ability to analyze performance trends across question types (eg, clinical scenarios, complexity, and format). Sixth, we focused on comparing OpenAI-o1 and GPT-4 and excluded human performance benchmarks (eg, from medical students) due to the study’s rapid initiation in October 2024, immediately following the release of OpenAI-o1. Given GPT-4’s extensive dataset training and OpenAI-o1’s enhanced reasoning capabilities, our primary objective was to promptly assess their differences in a medical context; frequent updates to LLMs and the time required for ethics approval and participant recruitment precluded human comparisons. Future research should integrate such comparisons. Lastly, we did not statistically evaluate the significance of the observed performance differences, further limiting our findings’ interpretability. The “black box” nature of both OpenAI-o1 and GPT-4 also limits interpretability; future research should use methods like attention analysis and causal reasoning tests and compare these models with open-source alternatives (eg, DeepSeek, Qwen) to enhance reproducibility and transparency.

The improved reasoning abilities of OpenAI-o1 may hold promise for real-world clinical applications. However, these findings are preliminary, and further research is needed to determine whether integrating such models into decision-support systems can contribute to reducing errors and enhancing patient care.

## Supplementary material

10.2196/68527 Multimedia Appendix 1 Responses of GPT-4 and OpenAI-o1 to Japanese National Medical Licensing Examination questions.

## Abbreviations

JNMLE: Japanese National Medical Licensing Examination
LLM: large language model
