# Supplementary material for: The Advanced Reasoning Capabilities of Large Language Models for Detecting Contraindicated Options in Medical Exams
Source: JMIR Med Inform. 2025 May 12;13:e68527. doi: 10.2196/68527 (PMC12088613; doi:10.2196/68527)
Supplement: Multimedia Appendix 1 [file medinform-v13-e68527-s001.docx]

**Appendix. Responses of ChatGPT-4 and OpenAI-o1 to Japanese National Medical Licensing Examination Questions.**

| **Japanese National Medical Licensing Exam Questions^a^** | **Responses of GPT-4**  **(Japanese / English)** | | **OpenAI-o1**  **(Japanese / English)** | |
| --- | --- | --- | --- | --- |
|  | **Choosing**  **Correct**  **Answers** | **Choosing**  **Contra-**  **indication** | **Choosing**  **Correct**  **Answers** | **Choosing**  **Contra-**  **indication** |
| **Q1.**  An unidentified middle-aged man was rescued from under the rubble of a building that collapsed during a magnitude 7 earthquake 30 minutes ago. There is no response when called. Respiratory rate is 32 per minute, shallow. Capillary refill time is 3 seconds. There is significant bleeding due to crush injuries on both lower legs. Extremities are notably cold. The disaster area is widespread, and there are many victims trapped under buildings. Which color of triage tag is appropriate for this patient?  a Green  b Yellow  c Red  d White  e Black | Correct /  Correct | Incorrect /  Incorrect | Correct /  Correct | Correct /  Correct |
| **Q2.**  A 19-year-old male. Visited the hospital for the investigation of leukocytosis. He presented to a local clinic one week prior with fever and sore throat, where he was noted to have leukocytosis and was referred for further evaluation. His temperature is 37.8°C. Pulse 92 per minute, regular. Blood pressure 118/76 mmHg. No abnormalities in the palpebral or bulbar conjunctivae. Both tonsils are red and swollen, with a white coating on the surface. Several tender lymph nodes up to 2 cm in diameter are palpable on both sides of the neck. No abnormalities in heart or lung sounds. The abdomen is flat and soft, with the liver palpable 2 cm below the costal margin. The spleen is not palpable. Blood findings: red blood cells 4.93 million, Hb 14.3 g/dL, Ht 44%, white blood cells 26,000 (band neutrophils 1%, segmented neutrophils 7%, eosinophils 0%, basophils 0%, monocytes 2%, lymphocytes 75%, atypical lymphocytes 15%), platelets 180,000. Biochemical findings: total bilirubin 1.3 mg/dL, direct bilirubin 0.7 mg/dL, AST 202 U/L, ALT 268 U/L, LD 637 U/L (normal 124-222), ALP 306 U/L (normal 38-113), creatinine 0.9 mg/dL, uric acid 7.1 mg/dL. CRP 0.9 mg/dL.  Which of the following is an appropriate management for this patient?  a. Watchful waiting  b. Plasma exchange  c. Administration of acyclovir  d. Administration of ampicillin  e. Administration of cytotoxic chemotherapy | Incorrect /  Correct | Correct /  Correct | Correct /  Correct | Correct /  Correct |
| **Q3.**  A 3-year-old boy, brought to the hospital by his mother due to difficulty breathing. He started having nocturnal cough a week ago, and since last night, his difficulty breathing worsened, prompting a visit to the emergency room. He is alert and oriented. Height 92 cm, weight 13 kg. Temperature 37.2°C. Seated pulse 120/min, regular. Blood pressure 110/78 mmHg. Respiratory rate 36/min. SpO₂ 96% (room air). Bilateral supraclavicular masses of 2 cm diameter are palpable. Heart sounds are normal, and stridor is heard in the chest. The abdomen is flat and soft, with no palpable liver or spleen. Blood findings: red blood cells 4.5 million, Hb 12.1 g/dL, Ht 38%, white blood cells 20,000 (blasts 14%, neutrophils 27%, eosinophils 3%, monocytes 6%, lymphocytes 50%), platelets 350,000. A chest X-ray shows a large mediastinal mass. Difficulty breathing and cyanosis rapidly appear in the supine position, with SpO₂ dropping to 74%. Returning to a seated position and administering oxygen at 10 L/min via mask improved SpO₂ to 98% and eased the breathing difficulty.  Which of the following is the appropriate management for this patient?  a. Thoracotomy  b. Endotracheal intubation  c. Thoracentesis  d. Inhalation of bronchodilators  e. Instruct to maintain sitting position and discharge home | Correct /  Correct | Incorrect /  Incorrect | Correct /  Correct | Incorrect /  Correct |
| **Q4.**  A 48-year-old man visited the clinic complaining of vision impairment in his right eye. While working without protective eyewear, a metal fragment entered his right eye, and subsequently, he became unable to see with that eye. Vision is hand motion in the right eye (not correctable) and 1.2 in the left eye (not correctable). Which of the following tests is useful for diagnosis?  a. Fluorescein angiography  b. Orbital plain CT scan  c. Electroretinography (ERG)  d. Ocular ultrasound  e. Orbital plain MRI | Correct /  Correct | Correct /  Correct | Correct /  Correct | Correct /  Correct |
| **Q5.**  Which of the following is a candidate for hepatic artery chemoembolization for hepatocellular carcinoma?  a. Liver function Child-Pugh A, maximum tumor diameter 4cm, number of tumors 4, no extrahepatic metastasis, no main portal vein occlusion  b. Liver function Child-Pugh A, maximum tumor diameter 4cm, number of tumors 4, with extrahepatic metastasis, no main portal vein occlusion  c. Liver function Child-Pugh B, maximum tumor diameter 4cm, number of tumors 4, no extrahepatic metastasis, main portal vein occlusion present  d. Liver function Child-Pugh B, maximum tumor diameter 4cm, number of tumors 4, with extrahepatic metastasis, no main portal vein occlusion  e. Liver function Child-Pugh C, maximum tumor diameter 4cm, number of tumors 4, no extrahepatic metastasis, no main portal vein occlusion | Correct /  Correct | Correct /  Correct | Correct /  Correct | Correct /  Correct |
| **Q6.**  A 74-year-old woman visited the clinic with complaints of right hip pain. She had undergone right total hip replacement surgery for osteoarthritis seven years ago. Since then, she had been progressing well until six months ago when right hip pain developed and gradually worsened, leading to difficulty walking. She is alert and oriented. Height 156 cm, weight 46 kg. Temperature 37.2°C. Pulse 84/min, regular. Blood pressure 132/72 mmHg. Swelling, warmth, and redness are observed in the right hip. Blood findings: red blood cells 3.7 million, Hb 10.8 g/dL, Ht 33%, white blood cells 12,700, platelets 300,000. Biochemical findings: total protein 7.4 g/dL, albumin 3.4 g/dL, total bilirubin 0.6 mg/dL, AST 17 U/L, ALT 8 U/L, LD 134 U/L (normal 120-245), ALP 144 U/L (normal 38-113), γ-GT 16 U/L (normal 8-50), amylase 70 U/L (normal 37-160), blood urea nitrogen 12 mg/dL, creatinine 0.7 mg/dL, blood sugar 90 mg/dL, Na 143 mEq/L, K 4.0 mEq/L, Cl 105 mEq/L. CRP 6.2 mg/dL. X-rays of both hips taken at the time of the visit revealed loosening of the right artificial hip joint.  Which of the following should be done next?  a. Intra-articular injection of corticosteroids  b. Continuous lower limb traction  c. Range of motion exercises  d. Joint fluid culture  e. Plaster cast immobilization | Correct /  Correct | Correct /  Correct | Correct /  Correct | Correct /  Correct |
| **Q7.**  A 20-year-old male presented to the clinic with palpitations and severe headaches as his chief complaints.  Past Medical History: He has occasionally experienced palpitations and headaches since he was 17 years old. Today, while helping a friend move furniture, he experienced intense palpitations and headaches, prompting him to seek medical attention.  Past Health Screenings: High blood pressure noted during a health screening at university entrance.  Lifestyle: University student. No history of smoking or drinking.  Family History: His father is being treated for hypertension.  Current Condition: Conscious and alert. Height 172 cm, weight 55 kg. Temperature 36.3°C. Pulse 132/min, regular. Blood pressure 192/110 mmHg. Respiratory rate 24/min. Notable sweating observed. Facial flushing present. Cold limbs. No abnormalities in the chest or abdomen.  Lab Findings:   - Urine: Protein (−), Sugar (−). - Blood: Red blood cells 4.63 million, Hb 13.2 g/dL, Ht 40%, white blood cells 5,800, platelets 220,000. - Biochemical: Total protein 8.8 g/dL, AST 24 U/L, ALT 14 U/L, LD 183 U/L (normal 120–245), blood urea nitrogen 17 mg/dL, creatinine 0.8 mg/dL, uric acid 7.2 mg/dL, blood sugar 101 mg/dL, Na 136 mEq/L, K 4.2 mEq/dL, Cl 100 mEq/L. CRP 1.2 mg/dL. - After admission, further tests revealed: TSH 1.76 μU/mL (normal 0.2–4.0), FT3 3.6 pg/mL (normal 2.3–4.3), FT4 1.4 ng/dL (normal 0.8–2.2), aldosterone 6 ng/dL (normal 5–10), plasma renin activity 2.0 ng/mL/hr (normal 1.2–2.5), adrenaline 120 pg/mL (normal below 100), noradrenaline 1,200 pg/mL (normal 100–450). Urinary VMA 18 mg/day (normal 1.3–5.1). An abdominal ultrasound revealed a 2 cm mass on the left side of the abdomen. Blood pressure was controlled with intravenous antihypertensive drugs.   Which of the following oral antihypertensive drugs should be administered first?  a. Alpha-blocker  b. Angiotensin-converting enzyme (ACE) inhibitor  c. Calcium channel blocker  d. Beta-blocker  e. Loop diuretic | Correct /  Correct | Correct /  Correct | Correct /  Correct | Correct /  Correct |
| **Q8.**  A 6-month-old female infant was brought to the clinic because she was not moving her left lower limb. Two days prior, she had a fever reaching 38°C and was diagnosed with pharyngitis at a nearby clinic, where she was prescribed acetaminophen suppositories. The next day, her mother noticed that she cried intensely when her diaper was being changed. No abnormalities were noted in the neonatal period. Height 68 cm, weight 7.2 kg. Temperature 37.8°C. Pulse 132/min, regular. Blood pressure 96/68 mmHg. Respiratory rate 14/min. There is no spontaneous movement of the left lower limb, and she cries when the left hip joint is moved passively. No range of motion restriction in the right hip joint. Blood tests: red blood cells 4.5 million, Hb 12.0 g/dL, Ht 38%, white blood cells 12,600 (band neutrophils 4%, segmented neutrophils 80%, eosinophils 1%, basophils 1%, monocytes 5%, lymphocytes 10%), platelets 260,000. CRP 15 mg/dL. Gram-positive cocci were observed in the gram stain of the fluid aspirated from the left hip joint.　Which of the following actions should be taken?  a. Oral NSAIDs  b. Incision and drainage of the left hip joint  c. Overhead traction of both lower limbs  d. Wearing a Rehme-Bügel brace  e. Corticosteroid injection into the left hip joint | Correct /  Correct | Correct /  Correct | Correct /  Correct | Correct /  Correct |
| **Q9.**  A 52-year-old man presented with complaints of lower abdominal fullness and inability to urinate. This morning, he slipped off a stepladder at home and bruised his perineum. At the time of consultation, bleeding was observed from the external urethral orifice. He was alert and conscious. His height was 168 cm and weight was 72 kg. Body temperature was 36.7°C. Pulse was 72 beats per minute, regular. Blood pressure was 124/84 mmHg. Respiratory rate was 20 per minute. He complained of spontaneous pain in the perineum, where subcutaneous swelling and tenderness were noted. The lower abdomen was distended. Blood findings: red blood cells 4.5 million, Hb 14.1 g/dL, Ht 42%, white blood cells 13,200, platelets 250,000. Biochemical findings in blood: total protein 7.5 g/dL, albumin 4.0 g/dL, total bilirubin 1.2 mg/dL, AST 23 U/L, ALT 22 U/L, LD 179 U/L (normal range 120-245), urea nitrogen 16 mg/dL, creatinine 0.7 mg/dL, uric acid 5.5 mg/dL, blood glucose 98 mg/dL, sodium 141 mEq/L, chloride 104 mEq/L, calcium 9.9 mg/dL. Abdominal X-ray showed no pelvic fracture. Pelvic CT revealed a hematoma in the perineum. Retrograde urethrography showed extravasation of contrast material outside the urethra at the membranous part, and the bladder was not visualized. Which of the following procedures is appropriate to perform first?  a. Perform hemodialysis.  b. Create a nephrostomy.  c. Create a cystostomy.  d. Place a ureteral stent.  e. Perform embolization of the external iliac artery. | Correct /  Correct | Incorrect /  Incorrect | Correct /  Correct | Incorrect /  Incorrect |
| **Q10.**  A 76-year-old man came to the hospital complaining of abdominal distension and pain. He was diagnosed with acute monocytic leukemia (FAB classification M5) three months ago and received several types of chemotherapy. However, he has not achieved remission to date. A week ago, he started experiencing lower back pain and took NSAIDs, but the effect was insufficient. Since last night, his abdominal pain worsened and he became unable to walk independently. He is conscious but appears to be in agony. His height is 171 cm and weight is 54 kg. Body temperature is 37.1°C. Pulse is 88 beats per minute, regular. Blood pressure is 118/78 mmHg. His conjunctiva is pale, suggesting anemia. A systolic ejection murmur can be heard on the right edge of the sternum. There are no subcutaneous hemorrhages on his limbs. Blood findings: red blood cells 2.82 million, Hb 8.0 g/dL, Ht 26%, white blood cells 52,400 (bone marrow blasts 74%, band neutrophils 2%, segmented neutrophils 12%, eosinophils 1%, basophils 1%, lymphocytes 10%), platelets 100,000. Biochemical findings in blood: total protein 5.1 g/dL, albumin 2.8 g/dL, total bilirubin 0.9 mg/dL, direct bilirubin 0.2 mg/dL, AST 34 U/L, ALT 37 U/L, LD 1,350 U/L (normal range 120-245), urea nitrogen 19 mg/dL, creatinine 1.3 mg/dL, uric acid 9.8 mg/dL. Abdominal ultrasound and CT scan showed extensive enlargement of the mesenteric and retroperitoneal lymph nodes, with suspected compression and infiltration of the gastrointestinal tract. Which of the following treatments should be considered first at this point?  a. Administration of albumin preparations  b. Administration of opioids  c. Platelet transfusion  d. Whole-body radiation therapy  e. Chemotherapy | Correct /  Correct | Correct /  Correct | Correct /  Correct | Correct /  Correct |
| **Q11.**  A 3-year-old girl was brought to the clinic by her grandfather, who was concerned that she was not moving her right elbow. She stopped moving her right elbow immediately after her 8-year-old brother pulled on her right hand while they were playing at home. Tenderness is observed around the radial head of the right elbow joint. There is no swelling, heat, or redness in the same area. No abnormalities are found in the shoulder and hand joints. No fractures are seen in the right elbow joint X-ray. Which of the following treatments is appropriate?  a. NSAIDs  b. Manual reduction  c. Splint fixation  d. Elbow joint capsulotomy  e. Elbow joint range of motion exercises | Correct /  Correct | Correct /  Correct | Correct /  Correct | Correct /  Correct |
| **Q12.**  A 72-year-old man came to the clinic complaining of lower abdominal pain.  Past medical history: He has been treated for hypertension for the past 10 years and is on antihypertensive medication. One month ago, he started experiencing palpitations with atrial fibrillation and was prescribed antiarrhythmic and anticoagulant drugs. Since yesterday afternoon, he has been unable to urinate, and his lower abdomen had been distended, but he waited to see how it would progress. This morning, he woke up with lower abdominal pain, which worsened, prompting him to seek medical attention.  Past medical history: Nothing notable. Lifestyle history: He has a smoking history of 20 cigarettes per day for 40 years. He drinks 1-2 glasses of sake per day. Family history: His father died of stomach cancer at the age of 68.  Current symptoms: He is conscious and alert. Height 165 cm, weight 61 kg. Body temperature 36.9°C. Pulse 52/min, irregular. Blood pressure 142/94 mmHg. Respiratory rate 18/min. SpO_2_ 96% (room air). No jugular venous distention observed. A II/VI systolic murmur is noted at the apex of the heart. No abnormalities in breath sounds. The abdomen is distended in the lower part, with tenderness observed. No edema in the lower legs. An abdominal ultrasound showed an enlarged bladder volume, and a decision was made to temporarily place a urinary catheter.  Which of the following is correct regarding catheter placement?  a. Place the patient in a lateral position during insertion.  b. Disinfect after applying lubricant to the penis.  c. Hold the penis vertically and gently lift it up.  d. Secure the urine collection bag at the same height as the bladder to the bedrail.  e. Inflate the balloon if there is strong resistance during insertion. | Correct /  Correct | Correct /  Correct | Correct /  Correct | Correct /  Correct |
| **Q13.**  Which of the following is the most appropriate treatment for a patient with psoriasis who is 10 weeks pregnant?  a. Oral PUVA therapy  b. Biological agent injection  c. Oral vitamin A derivative  d. Topical active vitamin D3  e. Topical corticosteroids | Incorrect /  Incorrect | Correct /  Correct | Correct /  Correct | Correct /  Correct |
| **Q14.**  A 60-year-old man came to the clinic after his wife noticed he had jaundice. He was diagnosed with diabetes at the age of 45 and has been taking oral diabetes medication. He is alert and conscious. Body temperature is 36.8°C. Pulse is 72 per minute, regular. Blood pressure is 128/76 mmHg. Respiratory rate is 14 per minute. His conjunctiva is mildly anemic, and there is yellowing of the sclera. No abnormalities are found in the heart or lung sounds. The abdomen is flat, and neither the liver nor spleen is palpable. There is mild tenderness in the right subcostal area. No edema is observed in the lower legs. Blood findings: red blood cells 3.56 million, Hb 10.8 g/dL, Ht 35%, white blood cells 7,500, platelets 380,000. Biochemical findings in blood: total protein 7.2 g/dL, albumin 4.2 g/dL, total bilirubin 5.8 mg/dL, direct bilirubin 3.7 mg/dL, AST 48 U/L, ALT 65 U/L, ALP 689 U/L (normal range 115-359), γ-GTP 243 U/L (normal range 8-50), urea nitrogen 45 mg/dL, creatinine 3.5 mg/dL, blood glucose 153 mg/dL, HbA1c 7.4% (normal range 4.6-6.2%). CRP 1.1 mg/dL.  Which of the following tests should be performed first?  a. Abdominal contrast-enhanced CT  b. Laparoscopic liver biopsy  c. Abdominal ultrasound  d. Magnetic resonance cholangiopancreatography (MRCP)  e. Endoscopic retrograde cholangiopancreatography (ERCP) | Incorrect /  Incorrect | Incorrect /  Incorrect | Correct /  Correct | Correct /  Correct |
| **Q15.**  A 21-year-old man came to the clinic with complaints of fever and cough. His body temperature is 39.2°C. Pulse is 108 per minute, regular. Blood pressure is 120/70 mmHg. Respiratory rate is 16 per minute. SpO_2_ is 97% (room air). Right after a blood sample was taken from the median cubital vein of the right elbow, he reported feeling unwell. He turned pale and started sweating all over, prompting an immediate cessation of the blood draw.  Which of the following actions should be taken next?  a. Place him in a supine position with legs elevated.  b. Apply a cold, wet compress to the puncture site.  c. Administer adrenaline intravenously.  d. Instruct him to increase his breathing rate.  e. Inject a local anesthetic subcutaneously at the puncture site. | Correct /  Correct | Correct /  Correct | Correct /  Correct | Correct /  Correct |
| **The total number of correct answers out of 15 questions** | 12 / 13 | 11 / 11 | 15 / 15 | 13 / 14 |

^a^We administered these questions to both GPT-4 and OpenAI-o1 in Japanese and English, respectively. The table displays the questions in English. The number of correct answers provided by each model was recorded. For the questions requiring the selection of contraindications, the following instruction was added: *Definition of contraindication: A contraindication is a specific situation in which a medicine, procedure, or surgery should not be used because it may be harmful to the person. Please select the answer that is a contraindicated option*
